# Operational research within the national tuberculosis control programme in Benin

**DOI:** 10.1186/s13104-017-2987-9

**Published:** 2017-11-29

**Authors:** Serge Ade, Dissou Affolabi, Mênonli Adjobimey, Gabriel Ade, Sévérin Anagonou, Ajay M. V. Kumar, Anthony D. Harries

**Affiliations:** 1 Faculté de Médecine, Université de Parakou, Parakou, Benin; 2 Programme National Contre la Tuberculose, 02BP: 8022, Cotonou, Benin; 3 International Union Against Tuberculosis and Lung Disease, Paris, France; 4 International Union Against Tuberculosis and Lung Disease, South-East Asia Office, New Delhi, India; 5 London School of Hygiene and Tropical Medicine, London, UK

**Keywords:** Benin, Operational research, Tuberculosis, Childhood TB, TB control programmes

## Abstract

**Objective:**

To document whether the placement of operational research (OR) fellows within disease control programmes in low and middle income countries leads to the implementation of operational research and improvements in policy and practice.

**Result:**

In 2012, an OR fellow was placed within the National TB Programme, Benin, to strengthen the implementation of operational research. From 2012 to 2015, eight OR projects were implemented, of which three contributed to changes in programme practice and five provided information which was not previously available from quarterly/annual reports. Two of these projects—one on the burden and treatment outcomes of childhood TB and one on tracing patients who had discontinued treatment—are discussed in more detail. OR should be strongly encouraged within national TB programme settings and an OR fellow facilitates this process.

## Introduction

In the last decade, there has been growing interest about operational research (OR). Various definitions of OR have been used, but one that is pragmatic and relevant at the field level is “research into interventions, strategies or tools that can enhance the quality, effectiveness, coverage or performance of disease control programmes or health systems in which the research is being conducted” [1]. Within National TB Programmes, operational research has been used to identify constraints that prevent objectives being met with the purpose of finding solutions and achieving those objectives [2]. Pillar three of the World Health Organization’s End TB Strategy emphasizes the need for intensified research and innovation to meet the goal of ending the tuberculosis epidemic and explicitly states that research should be undertaken to optimize implementation and impact [3].

In Bénin, a West-African country with about 10 million inhabitants [4], the National Tuberculosis Programme (NTP), started in 1978, provides free diagnostic and treatment services for the population. An OR unit was created within the NTP in 2010 with a view to scaling up programmatic operational research. Initially, the unit held monthly meetings lasting 1–2 h at which a research project or a specific area of OR was discussed. However, there was a lack of skilled and trained personnel to implement OR projects. In 2012, a part-time OR fellow, supported from the Centre for Operational Research, The International Union Against Tuberculosis and Lung Disease, Paris, France, was appointed within the NTP to promote and lead a TB-specific OR agenda. After undergoing a formal training in the Union-MSF run SORT IT Course [5], he has been working with NTP colleagues to develop and implement a TB-specific OR agenda in Benin and mentoring participants attending other SORT IT courses from around the region. We describe the operational research output within the NTP, Benin, since the OR fellow was appointed and outline how the research has informed policy and/or practice.

## Main text

### Aspect of interest

From 2012 to 2015, eight projects were developed, implemented and completed by the OR department with direction and support of the OR fellow. The titles, publication status, policy and practice implications of these eight projects are shown in Table 1. Two examples are illustrated in more detail below.

**Table 1:** Published research papers co-authored by the Benin operational research fellow, January 2012–December 2015

Year	Journal	Title of the paper	First author; volume number; page numbers
2013	Public health action	The burden and outcomes of childhood tuberculosis in Cotonou, Benin^a^	Ade S; vol 314; no 1; 15–19
2013	Public health action	MDR-TB treatment needs in patients previously treated for TB in Cotonou, Benin^b^	Ade S; vol 3; no 2; 160–165
2013	Transactions of the royal society of tropical medicine and hygiene	National profile and treatment outcomes of adult smear-negative pulmonary TB patients in Benin^b^	Ade S; 10.1093/trstmh/trt092
2014	PLoS One	National profile and treatment outcomes of patients with extrapulmonary tuberculosis in Benin^a^	Ade S; vol 9; no 4; e95603
2015	Public health action	Low prevalence of diabetes mellitus in patients with tuberculosis in Cotonou, Benin^b^	Ade S; vol 5; no 2; 147–149
2016	BMC health services research	Follow-up and tracing of tuberculosis patients who fail to attend their scheduled appointments in Cotonou, Benin: a retrospective cohort study^a^	Ade S; vol 5; no 2; 7 pages; 10.1186/s12913-015-1219-z
2016	Tuberculosis research and treatment	Characteristics and treatment outcomes of retreatment tuberculosis patients in Benin^b^	Ade S; vol 2016, Article ID 1468631, 7 pages http://dx.doi.org/10.1155/2016/1468631
2016	Tuberculosis research and treatment	Tuberculosis case finding in Benin, 2000–2014 and beyond: a retrospective cohort and Time Series Study^b^	Ade S; volume 2016, Article ID 3205843, 9 pages http://dx.doi.org/10.1155/2016/3205843

^a^Contributed to changes in practice

^b^Contributed to providing data and information that were not previously available

One of the first projects focused on the burden and outcomes of childhood TB in Cotonou, the capital city [6]. One of the NTP objectives was to increase TB case detection in the general population, especially among children aged ≤ 14 years. The quarterly aggregate data, collected at national level, did not provide sufficient information about the burden or management of childhood TB in the country. Furthermore, it was believed that some children treated for TB in specialist pediatric services were not notified to the NTP. Nothing was known about childhood treatment outcomes. The results of the OR study found that 16% of children diagnosed with TB in the general hospital were not notified to the NTP and treatment success was lower in children compared to adults (72% versus 90%) [7]. These findings led to an important meeting between NTP staff and pediatricians in Benin, where pediatricians were sensitized about the need to notify all TB cases and it was decided to produce a national guidebook for the management of childhood TB. This has been published and wide dissemination and training of health care providers on its contents is currently taking place. These measures will hopefully improve the management and treatment success among children with TB. We plan to assess this in a follow-up operational research study.

A second example focused on the follow-up and tracing of TB patients who failed to attend their scheduled appointments in Cotonou [8]. Since 2009, the NTP has tried to achieve a treatment success of 90% or more amongst new bacteriologically confirmed TB patients, and a unit was established with the responsibility of tracing patients who discontinued treatment in Cotonou. The NTP was interested to know whether investment in this unit was worthwhile. The OR found that 8% of patients missed one or more of their appointments, with tracing activities increasing the treatment success by a mere 4%. These findings suggested that other activities were needed to improve outcomes that included better quality counseling/education by health care workers before and during treatment, providing patients with sufficient quantities of medication in case of travel outside the country and keeping a register listing scheduled patient visits so that tracing activities can be started much earlier. Since the register has been established, the time taken before starting the tracing of poorly adherent patients has significantly reduced (unpublished data).

### Discussion

It is only in the last 5 years that a part-time OR fellow has been placed within the NTP in Benin. This has facilitated implementation and completion of relevant OR projects that have benefited the NTP; What has been achieved is in line with experience gained in Malawi, Central Africa, and in India [2, 9]. While some projects contributed to changes in programme practice, others provided useful information for programme planning and evaluation. Despite this success, more needs to be done in terms of building a critical mass of skilled personnel capable of pragmatically engaging in regular OR planning meetings and of expanding and completing a broader range of beneficial OR projects which can impact policy and practice [10]. For this purpose, three additional health workers have currently been trained on OR project implementation through the West African Regional Network for TB training which is built on the SORT-IT model.

The NTP in Benin has recently defined its research priorities in line with the WHO End TB Strategy with projects focusing on: (i) increasing TB case finding in both the general population and in vulnerable/at risk groups; (ii) strategies to reduce the burden of death in patients with TB and HIV-associated TB; and (iii) improving case detection in childhood TB. With an OR fellow in place and additional health workers trained in OR skills, we hope that these research priorities are implemented and the results will inform program decision-making.

### Limitations

There were three main limitations. First, the two studies that were reported in this paper were retrospective in nature and therefore constrained by the accuracy or not of the routinely collected data. Second, the actions that were implemented after the projects were completed have not yet been formally assessed or published in peer reviews papers. Third, the article is focused on TB which may not be of interest to those working in other disease control programmes.

## Abbreviations

OR: operational research
TB: tuberculosis
NTP: National Tuberculosis Programme
SORT-IT: Structured Operational Research and Training IniTiative
HIV: human immunodeficiency virus

## References

[1] Zachariah R, Harries AD, Ishikawa N, Rieder HL, Bissell K, Laserson K (2009). Operational research in low-income countries: what, why, and how. Lancet Infect Dis.

[2] World Health Organization (1999). TB research: putting research into policy and practice: the experience of the Malawi national tuberculosis programme.

[3] World Health Organization. Factsheet: Post-2015 Global TB Strategy and targets. World Health Organization; 2015. http://www.who.int/tb/post2015_TBstrategy.pdf?ua=1. Accessed 27 Nov 2016.

[4] Institut National de la Statistique et de l’Analyse Economique, Benin en chiffres. http://www.insae-bj.org. Accessed 27 Nov 2016.

[5] Ramsay A, Harries AD, Zachariah R, Bissell K, Hinderaker SG, Edginton M (2014). The structured operational research and training initiative for public health programmes. Public Health Action.

[6] Ade S, Harries AD, Trébucq A, Hinderaker SG, Ade G, Agodokpessi G (2013). The burden and outcomes of childhood tuberculosis in Cotonou, Benin. Public Health Action.

[7] World Health Organization (2015). Global tuberculosis report 2015.

[8] Ade S, Trébucq A, Harries AD, Ade G, Agodokpessi G, Wachinou P (2016). Follow-up and tracing of tuberculosis patients who fail to attend their scheduled appointments in Cotonou, Benin: a retrospective cohort study. BMC Health Serv Res.

[9] Kumar AMV, Satyanarayana S, Dar Berger S, Chadha SS, Singh RJ, Lal P (2015). Promoting operational research through fellowships: a case study from the South-East Asia Union Office. PHA.

[10] Zachariah R, Guillerm N, Berger S, Kumar AM, Satyanarayana S, Bissell K (2014). Research to policy and practice change: is capacity building in operational research delivering the goods?. Trop Med Int Health.

